# Secreted phospholipase A2 of *Clonorchis sinensis* activates hepatic stellate cells through a pathway involving JNK signalling

**DOI:** 10.1186/s13071-017-2082-z

**Published:** 2017-03-16

**Authors:** Yinjuan Wu, Ye Li, Mei Shang, Yu Jian, Caiqin Wang, Adham Sameer A. Bardeesi, Zhaolei Li, Tingjin Chen, Lu Zhao, Lina Zhou, Ai He, Yan Huang, Zhiyue Lv, Xinbing Yu, Xuerong Li

**Affiliations:** 1 Department of Parasitology, Zhongshan School of Medicine, Sun Yat-sen University, Guangzhou, Guangdong 510080 People’s Republic of China; 2 Key Laboratory of Tropical Disease Control (Sun Yat-sen University), Chinese Ministry of Education, Guangzhou, Guangdong 510080 People’s Republic of China; 3 Provincial Engineering Technology Research Center for Biological Vector Control, Guangzhou, Guangdong 510080 People’s Republic of China

**Keywords:** Phospholipase A2, *Clonorchis sinensis*, Enzyme, Liver fibrosis, Cholangiocarcinoma, JNK signalling pathway

## Abstract

**Background:**

Secreted phospholipase A2 (sPLA2) is a protein secreted by *Clonorchis sinensis* and is a component of excretory and secretory products (*Cs*ESPs). Phospholipase A2 is well known for its role in liver fibrosis and inhibition of tumour cells. The JNK signalling pathway is involved in hepatic stellate cells (HSCs) activation. Blocking JNK activity with SP600125 inhibits HSCs activation. In a previous study, the protein *Cs*sPLA2 was expressed in insoluble inclusion bodies. Therefore, it’s necessary to express *Cs*sPLA2 in water-soluble form and determine whether the enzymatic activity of *Cs*sPLA2 or cell signalling pathways is involved in liver fibrosis caused by clonorchiasis.

**Methods:**

*Balb*/C mice were given an abdominal injection of MBP-*Cs*sPLA2. Liver sections with HE and Masson staining were observed to detect accumulation of collagen. Western blot of mouse liver was done to detect the activation of JNK signalling pathway. In vitro, HSCs were incubated with MBP-*Cs*sPLA2 to detect the activation of HSCs as well as the activation of JNK signalling pathway. The mutant of MBP-*Cs*sPLA2 without enzymatic activity was constructed and was also incubated with HSCs to check whether activation of the HSCs was related to the enzymatic activity of MBP-*Cs*sPLA2.

**Results:**

The recombinant protein MBP-*Cs*sPLA2 was expressed soluble and of good enzymatic activity. A mutant of *Cs*sPLA2, without enzymatic activity, was also constructed. In vivo liver sections of *Balb*/C mice that were given an abdominal injection of 50 μg/ml MBP-*Cs*sPLA2 showed an obvious accumulation of collagen and a clear band of P-JNK1 could be seen by western blot of the liver tissue. In vitro*,* MBP-*Cs*sPLA2, as well as the mutant, was incubated with HSCs and it was proved that activation of HSCs was related to activation of the JNK signalling pathway instead of the enzymatic activity of MBP-*Cs*sPLA2.

**Conclusions:**

Activation of HSCs by *Cs*sPLA2 is related to the activation of the JNK signalling pathway instead of the enzymatic activity of *Cs*sPLA2. This finding could provide a promising treatment strategy to interrupt the process of liver fibrosis caused by clonorchiasis.

## Background

*Clonorchis sinensis* is a parasite prevailing mainly in eastern countries. In China, northern Vietnam and South Korea about 15 million people are infected; 13 million infected people come from China alone [1–4]. In 1975, eggs of *C. sinensis* were found in a corpse of the Western Han Dynasty in Hubei province, which showed that the disease appeared at least two millennia ago [5]. When raw or undercooked infected fish is eaten by people, the metacercaria is excysted by gastric juice from the flesh in the duodenum [6]. Cholangitis, cholecystitis and cholelithiasis are the main complications caused by the chronic infection with C. *sinensis* [7–9]. Infiltration of eosinophils and mast cells, fibrosis and mucosal hyperplasia of the gallbladder wall are also associated with infection [10]. Infection is widely considered to be related to cholangiocarcinoma (CCA). The morbidity of CCA caused by *C. sinensis* each year is about 0.035% in males and 0.025% in females [2].

*Clonorchis sinensis* excretory/secretory products (*Cs*ESPs) is proved to result in liver fibrosis in clonorchiasis. Secreted phospholipase A2 (sPLA2) is a protein secreted by *C. sinensis* and is a component of *Cs*ESPs. sPLA2 enzymes, characterised by low-molecular-weight, calcium-dependent and a His-Asp catalytic dyad, refer to the largest family among the phospholipase A2 (PLA2) superfamily [11]. The parasite lives in the bile duct of the liver and is exposed to bile which is rich in fatty acid*. Cs*sPLA2, which was proved to be a component of the ESPs of *C. sinensis*, plays an important role in the growth of the parasite by breaking down complex lipids [12, 13]. Liver fibrosis is observed in patients with moderate or severe infection of liver fluke. Deficiency of Group IVA phospholipase A2 (IVA-PLA2) alleviates the deposition of lipids and delays the formation of liver fibrosis [14]. *Cs*sPLA2 of *C. sinensis* may lead to liver fibrosis and HSCs activation which is related to c-Jun N-terminal kinase (JNK) signalling pathway. When JNK signalling pathway is blocked by inhibitor SP600125, hepatic stellate cells (HSCs) activation is inhibited, with less proliferation and reduced expression of α-smooth muscle actin (α-SMA) [15]. An increased expression of collagen III has been detected in LX-2 cells by quantitative RT-PCR after incubating with the recombinant *Cs*sPLA2 which was a renatured protein from inclusion bodies [12]. *Cs*sPLA2 was difficult to express in soluble form and only little insoluble inclusion bodies renatured by dilution and dialysis were obtained. Both of cell cycle analysis and MTT test of LX-2 demonstrated that the percentage of cells in the proliferation phase was higher [12]. PLA2 from snake venoms have been widely studied because of the pathophysiological and pharmacological effects including antiangiogenic and antitumor properties on living organisms [16–18].

To detect the activation of hepatic stellate cells LX-2 as well as activation of the JNK signalling pathway by the recombinant protein MBP-*Cs*sPLA2, the recombinant protein MBP-*Cs*sPLA2 was expressed in soluble form in *E. coli* and displayed the phospholipase activity. MBP-*Cs*sPLA2 could activate hepatic stellate cells which could lead to liver fibrosis. The mechanism of the hepatic stellate cells activation is involved in the activation of JNK signalling pathway instead of the enzymatic activity of the protein, which could provide a promising strategy to interrupt the process of liver fibrosis caused by infection of *Clonorchis sinensis*.

## Methods

### Cloning, expression, and purification of the recombinant protein MBP-*Cs*sPLA2

Polymerase chain reaction (PCR) was used to amplify CDS but not the signal peptide sequence of *Cs*sPLA2 gene (GenBank Accession no. DQ974199). The forward and reverse oligonucleotide primers were: 5′-CTA GTC TAG AAA ACC ACG GTC AAT TTC A-3′ and 5′-GGG AAG CTT GCT CAT ACA GTA ATG TAC G-3′ with *Xba* I and *Hind* III sites to the 5' ends, respectively (underlined). *Clonorchis sinensis* adult cDNA was used as template. Total RNAs from adult worms were extracted in Trizol reagent (Invitrogen, Carlsbad, USA) Amplicons were cloned into pMAL-c2X (New England Biolabs, Ipswich, USA). The nucleotide sequences of the recombinant plasmid *Cs*sPLA2/pMAL-c2X were confirmed by DNA sequencing. The recombinant plasmid *Cs*sPLA2/pMAL-c2X was expressed in *E. coli* BL21 (DE3) in Luria-Bertani medium containing 50 μg/ml ampicillin. The final concentration of 0.3 mM isopropyl-1-thio-galactoside (IPTG) was added to induce expression, and the culture was further incubated at 37 °C for 4 h. The MBP-*Cs*sPLA2 fusion protein was purified by amylose resin (New England Biolabs, Ipswich, USA) MBP-*Cs*sPLA2 was further purified by anion exchange chromatography with an anion exchange column (HiTrap Q FF).

### SDS-PAGE and western blot

The recombinant protein MBP-*Cs*sPLA2 was subjected to SDS-PAGE (12% polyacrylamide gel) and immobilized onto polyvinylidenedifluoride membrane. The membrane was incubated with the primary antibodies (*Cs*sPLA2 monoclonal antibody, 1:3000 dilutions in 1% BSA-PBS) overnight at 4 °C after blocking with 5% skim milk at room temperature for 2 h. Then the membrane was washed and incubated with goat anti-mouse IgG conjugated HRP (1:2000 dilution) (Proteintech, Chicago, USA) at room temperature for 1 h. The results were visualized by chemiluminescence (chemiluminescent HRP substrate, Millipore, Billerica, USA)

### Mass spectrometry

The mass spectrometry identification of the recombinant protein MBP-*Cs*sPLA2 was carried out by the Proteomics Center, Zhongshan School of Medicine, Sun Yat-sen University.

### Enzyme activity assay

The enzyme activity of CssPLA2 was detected by sPLA2 Assay Kit (Cayman, Ann Arbor, USA). The recombinant protein MBP-*Cs*sPLA2 was dialysed against PBS. Bee Venom PLA2 was used as the positive and as a negative control, the MBP protein expressed by empty plasmid pMAL-c2x was used. 10 μl DTNB, 10 μl sample and 5 μl assay buffer were added to sample wells. 10 μl DTNB, 10 μl Bee Venom PLA2 and 5 μl assay buffer were added to positive control wells. Negative control wells contain 10 μl of DTNB, 10 μl of MBP and 5 μl of assay buffer. The reactions were initiated by adding 200 μl substrate solution to all the wells. The plate was carefully shaken and read at an absorbance of 414 nm every 1 min using a microplate spectrophotometer (SpectraMax M5, American Molecular Devices, Sunnyvale, USA).

### Western blot of mouse liver tissue to detect the activation of the JNK signalling pathway

*Balb*/C mice with weights of 18–20 g, 6–8 weeks of age, male and cared for in a SPF environment were divided into five groups with three mice in one group, and were given an abdominal injection of PBS, 100 μg ESPs of *Clonorchis sinensis*, 100 μg MBP protein (negative control), 50 μg of MBP-CsPLA2 protein and 100 μg of MBP-*Cs*sPLA2 protein, respectively, twice a week. The mice were sacrificed four weeks later and liver sections with HE and Masson staining were observed for collagen accumulation. Western blot of the mice liver tissue was also done to detect the activation of the JNK signalling pathway related to liver fibrosis.

### Construction of MBP-*Cs*sPLA2 mutant without enzymatic activity

The enzymatic center of PLA2 protein is comprised of two key amino acids, His192 and Asp222. Here, His192 of *Cs*sPLA2 was changed into Asn192. Fast mutagenesis system (Transgen Biotech, Beijing, China) was applied to deliver the mutation. PCR amplification was performed with two overlapping primers. Both primers contain the target mutation. The forward and reverse oligonucleotide primers were: 5′-CTG ACA TGT GCT GTC GAA CTA ATG ACC GAT G-3′ and 5′-TAG TTC GAC AGC ACA TGT CAG TCT CGA TTT C-3′. PCR was performed under conditions of the 20 s at 94 °C, 20 s at 57 °C, and 3.5 min at 72 °C for 25 cycles and recombinant plasmid *Cs*sPLA2/pMAL-c2X was used as a template. The difference between the mutant and MBP-*Cs*sPLA2 is only one amino acid, therefore the expression and purification of the protein of mutant are the same with that of MBP-*Cs*sPLA2. The enzymatic activity of the mutant is determined by sPLA2 Assay Kit (Cayman, Ann Arbor, USA).

### Detection of collagen I in LX-2 cells incubated with MBP-*Cs*sPLA2 by ELISA

LX-2 cells were cultured in Dulbecco’s modified Eagle medium (DMEM, Gibco, Carlsbad, USA) with 10% fetal bovine serum (FBS), penicillin (100 U/ml) and streptomycin (100 μg/ml) at 37 °C under 5% CO_2_. The effect of the recombinant MBP-*Cs*sPLA2 on the production of collagen I by LX-2 cells was assessed by ELISA. Cells were removed from the cell-culture plastic bottle by trypsin and seeded into 6-well culture plates (6,000 cells/well, Nest, Wuxi, China) in the presence of DMEM containing 10% FBS for 24 h. Diluted in DMEM with 2% FBS, 25 μg/ml ESPs, 25 μg/ml MBP, 25 μg/ml MBP-*Cs*sPLA2 and PBS were included with cells. After 36 h or 48 h, the supernatant of cells was detected by ELISA. Collagen I rabbit antibody (1:2,000 dilutions, Abcam, London, UK) was used as the first antibody and HRP-conjugated goat anti-rabbit IgG was used as the secondary antibody (1:10,000 dilutions, Proteintech, Chicago, USA). Each well was incubated with tetramethylbenzidine (TMB) solution. Finally, the reaction was stopped by 2 M H_2_SO_4_, and the absorbance of each well was detected at 450 nm.

### Detection of α-SMA in LX-2 cells by Western blotting

The effects of the recombinant MBP-*Cs*sPLA2 on LX-2 activation were assessed by Western blot. The expression of activation markers normalized by *β*-actin was detected in LX-2, which were divided into three groups and incubated with 25 μg/ml MBP, 25 μg/ml MBP-*Cs*sPLA2 and PBS, respectively, for 48 h. To further investigate the dose dependence of MBP-*Cs*sPLA2 and the effect of the JNK inhibitor in the process, LX-2 cells were then divided into five groups and incubated with 25 μg/ml MBP, 10 μg/ml MBP-*Cs*sPLA2, 25 μg/ml MBP-*Cs*sPLA2, PBS and 25 μg/ml MBP-*Cs*sPLA2 together with 5 μM JNK inhibitor (SP600125), respectively, for 48 h. Then all the cells were lysed by cell lysing buffer and concentration of protein was detected by bicinchoninic acid (BCA) protein assay kit (Novagen, Darmstadt, Germany). The recombinant protein MBP-*Cs*sPLA2 was subjected to SDS-PAGE (12% polyacrylamide gel) and immobilized onto a polyvinylidenedifluoride membrane. The membrane was incubated with the primary antibodies (α-SMA antibody, 1:500 dilutions in 1% BSA-PBS) (Proteintech, Chicago, USA) overnight at 4 °C after blocking with 5% skim milk at room temperature for 2 h. Then the membrane was washed and incubated with goat anti-rabbit immunoglobulin G conjugated HRP (1:5,000 dilution) (Proteintech, Chicago, USA) at room temperature for 1 h. The results were visualized by chemiluminescence (chemiluminescent HRP substrate, Milipore, Billerica, USA).

### Quantitative RT-PCR analysis of activation markers of LX-2

LX-2 cells were incubated with 25 μg/ml MBP-*Cs*sPLA2, 25 μg/ml mutant and 25 μg/ml MBP-*Cs*sPLA2 together with 5 μM JNK inhibitor (SP600125), respectively, for 24 h. Total RNA was extracted from LX-2 cells using Trizol. One microgram of total RNA was reverse transcribed into cDNA with the reverse transcriptase kit (Takara, Dalian, China). Using SYBR Premix ExTaq, cDNA was used as the template for PCR amplification. The primers of human smooth muscle actin (α-SMA) were 5′-CCA GGG CTG TTT TCC CAT CC-3′ (forward primer) and 5′-GCT CTG TGC TTC GTC ACC CA-3′ (reverse primer). The primers of human collagen III were 5′-GGT CCT CCT GGA ACT GCC GGA-3′ (forward primer) and 5′-GAG GAC CTT GAG CAC CAG CGT GT-3′ (reverse primer). The primers of human *β*-actin were 5′-GTC CAC CGC AAA TGC TTC TA-3′ (forward primer) and 5′-TGC TCT CAC CTT CAC CGT TC-3′ (reverse primer). The human *β*-actin served as the internal standard. PCR conditions were as follows: 95 °C for 30 s, 40 cycles of 95 °C for 5 s, and 60 °C for 20 s, with an incremental increase of 0.1 °C/s from 60 to 95 °C.

### Detection of P-JNK1 in LX-2 cells incubated with MBP-*Cs*sPLA2 and the mutant respectively by Western blotting

The effects of the recombinant MBP-*Cs*sPLA2 and the mutant on JNK signalling activation in LX-2 cells was assessed by Western blot. LX-2 cells were incubated for 48 h with 25 μg/ml MBP, 25 μg/ml MBP-*Cs*sPLA2, 25 μg/ml mutant and PBS, respectively. The expression of phospho-JNK1 was detected and normalized by *β*-actin in LX-2 cells. Then all the cells were lysed by cell lysing buffer and concentration of protein was detected by bicinchoninic acid (BCA) protein assay kit (Novagen). The protein was subjected to SDS-PAGE (12% polyacrylamide gel) and immobilized onto a polyvinylidenedifluoride membrane. The membrane was incubated with the primary antibodies (P-JNK1 antibody, 1:1000 dilutions in 1% BSA-PBS) (CST, Boston, USA) overnight at 4 °C after blocking with 5% skim milk at room temperature for 2 h. Then the membrane was washed and incubated with goat anti-rabbit immunoglobulin G conjugated HRP (1:5000 dilution) (Proteintech, Chicago, USA) at room temperature for 1 h. The results were visualized by chemiluminescence (chemiluminescent HRP substrate, Millipore, Billerica, USA)).

### Statistical analysis

Results were analyzed by the software GraphPad Prism 5 using the method *t*-test to compare the difference between the experimental group and the control group, considering values of *P* < 0.05 as significant.

## Results

### Prokaryotic expression and purification of MBP-*Cs*sPLA2

**Fig. 1:**
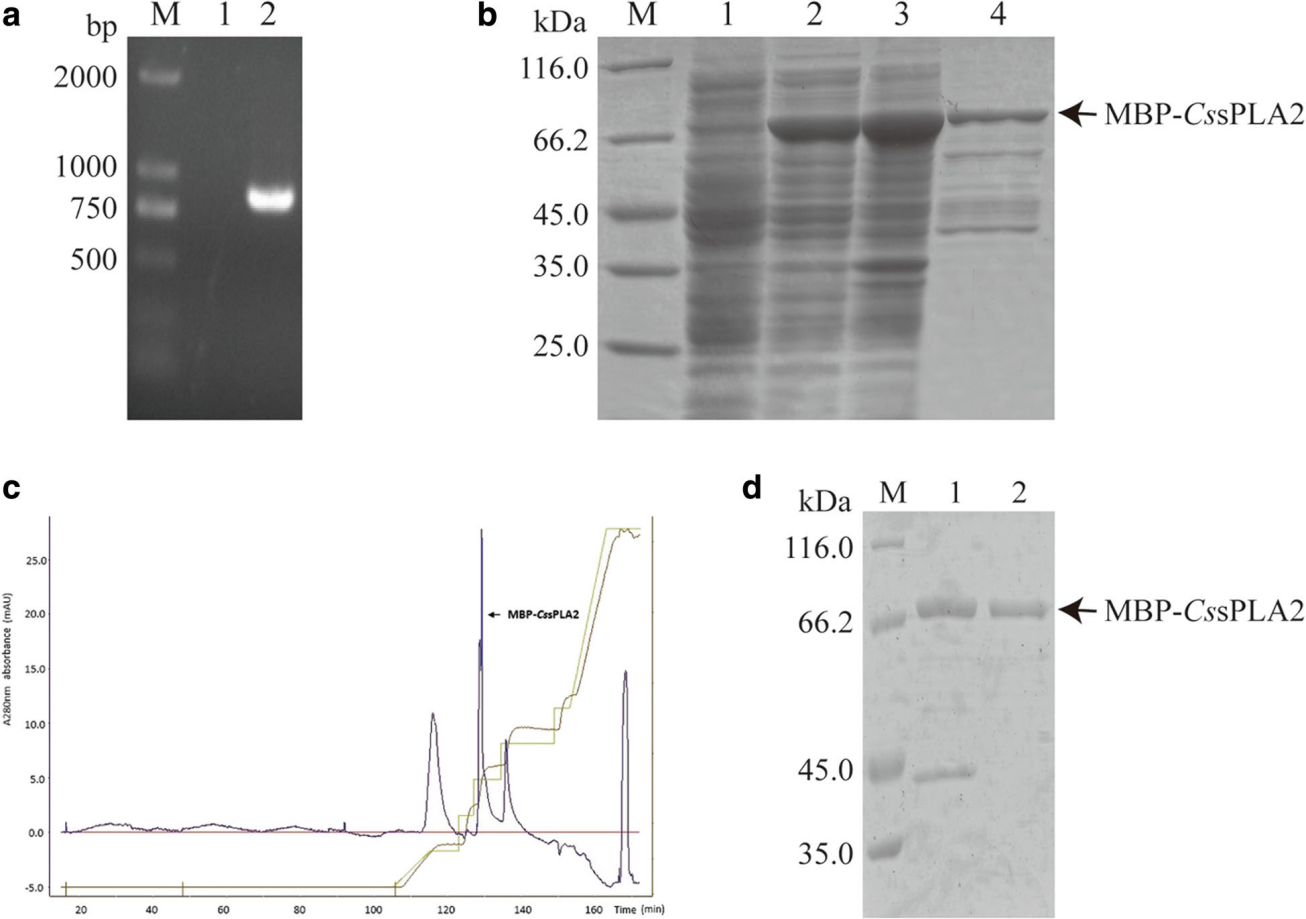
Prokaryotic expression and purification of protein *Cs*sPLA2 with plasmid pMAL-c2X. **a** PCR product of *Cs*sPLA2 (828 bp). Lane M: DNA molecular marker; Lane 1: ddH2O negative control; Lane 2: PCR product of *Cs*sPLA2 (828 bp). **b** SDS-PAGE analysis of the prokaryotic expression product of *Cs*sPLA2. Lane M: protein molecular weight marker; Lane 1: *Cs*sPLA2/pMAL-c2X transformants without IPTG induction; Lane 2: supernatant of the lysate of bacteria with *Cs*sPLA2/pMAL-c2X after IPTG induction; Lane 3: precipitation of the lysate of bacteria with *Cs*sPLA2/pMAL-c2X after IPTG induction; Lane 4: purified MBP-*Cs*sPLA2 protein. **c** Anion exchange chromatography for purification of MBP-*Cs*sPLA2 protein. **d** SDS-PAGE analysis of MBP-*Cs*sPLA2 protein after anion exchange chromatography. Lane M: protein molecular weight marker; Lane 1: MBP-*Cs*sPLA2 protein purified by amylose resin; Lane 2: MBP-*Cs*sPLA2 protein purified by amylose resin and anion exchange chromatography

**Fig. 2:**
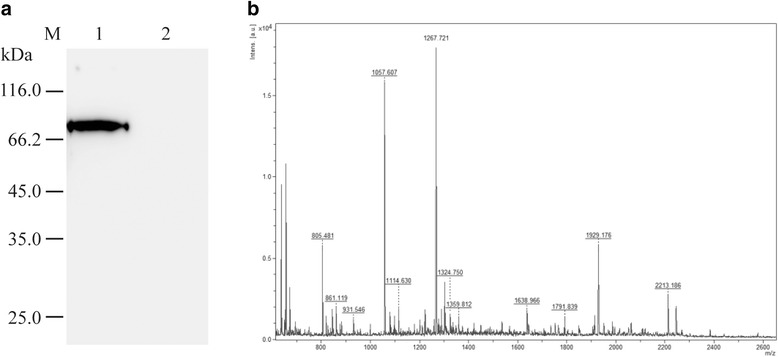
Identification of protein MBP-*Cs*sPLA2 by western blot and mass spectrum. **a** The recombinant protein MBP-*Cs*sPLA2 was identified by western blot with *Cs*sPLA2 monoclonal antibody. *Cs*sPLA2 monoclonal antibody (1:3,000 dilution) was used as first antibody and goat anti-mouse immunoglobulin G conjugated HRP (1:2000 dilution) was used as the second antibody. Lane 1: the recombinant protein MBP-*Cs*sPLA2; Lane 2: protein MBP. **b** The recombinant protein MBP-*Cs*sPLA2 was identified by mass spectrometry

The length of PCR product of *Cs*sPLA2 was 828 bp (Fig. 1a). The soluble MBP-*Cs*sPLA2 was expressed with a MBP tag in *E. coli* BL21 (DE3) after being induced with 0.3 mM IPTG at 37 °C for 4 h. The recombinant protein was purified by amylose resin (Fig. 1b) and anion exchange chromatography (Fig. 1c). The purified MBP fusion protein showed a single band with a molecular mass around 76 kDa in 12% SDS-PAGE, consistent with the predicted molecular mass (Fig. 1d). The protein MBP-*Cs*sPLA2 was identified by both western blot with the *Cs*sPLA2 monoclonal antibody (Fig. 2a) and mass spectrometry (Fig. 2b). The final protein concentration was 0.5 mg/ml while the concentration of endotoxin-free MBP-*Cs*PLA2 was about 0.2 mg/ml.

### Enzymatic activity of MBP-*Cs*sPLA2

**Fig. 3:**
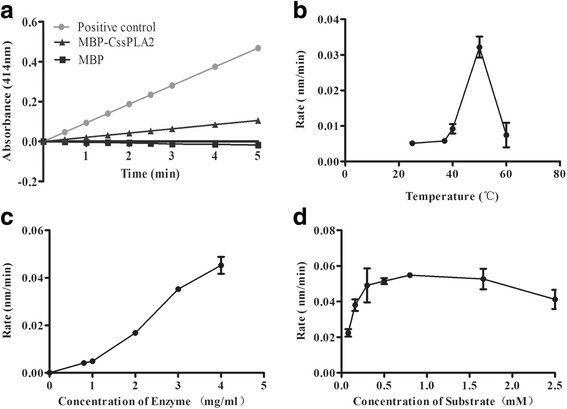
Detection of the enzymatic activity of MBP-*Cs*sPLA2. The enzyme activity of CssPLA2 was detected by sPLA2 Assay Kit. Bee venom PLA2 was used as positive controls and the MBP protein expressed by empty plasmid pMAL-c2X was used as negative control. **a** Detection of enzymatic activity of MBP-*Cs*sPLA2. **b** Influence of the temperature on the enzymatic activity of MBP-*Cs*sPLA2. **c** Influence of enzyme concentration to enzymatic activity of MBP-*Cs*sPLA2. **d** Influence of the substrate concentration on the enzymatic activity of MBP-*Cs*sPLA2

The enzymatic activity of MBP-*Cs*sPLA2 was detected with sPLA2 Assay Kit, it showed good enzymatic activity with protein MBP as a negative control and bee venom sPLA2 as a positive control (Fig. 3a). The enzyme was heat resistant and showed good enzymatic activity at 50 °C (Fig. 3b). The enzymatic activity got better with either higher concentration of enzyme or higher concentration of substrate until an optimum concentration was obtained. (Fig. 3c, d).

### Liver fibrosis and activation of the JNK signalling pathway by MBP-*Cs*sPLA2 in liver tissue of *Balb*/C mouse

**Fig. 4:**
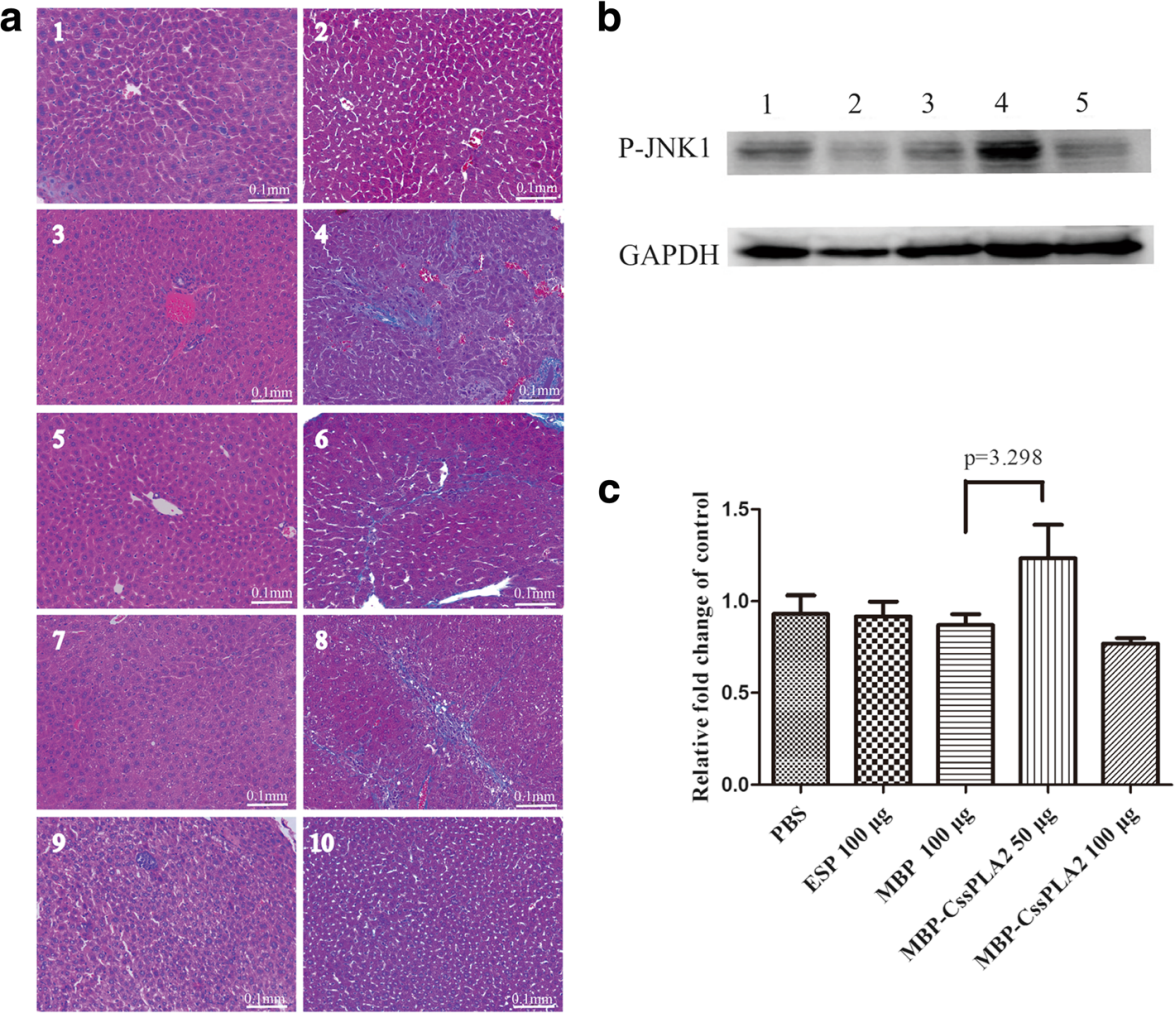
JNK signalling was activated in the liver tissue of *Balb*/C mouse with abdominal injection of MBP-*Cs*sPLA2. **a** Liver sections of five groups of *Balb*/C mouse with HE and Masson staining. *Sections 1 & 2*, HE and MASSON staining of liver sections of *Balb*/C mouse with an abdominal injection of PBS; *Sections 3 & 4*, HE and MASSON staining of liver sections of *Balb*/C mouse with an abdominal injection of 100 μg ESPs; *Sections 5 & 6*, HE and MASSON staining of liver sections of *Balb*/C mouse with an abdominal injection of 100 μg MBP; *Sections 7 & 8*: HE and MASSON staining of liver sections of *Balb*/C mouse with an abdominal injection of 50 μg MBP-*Cs*sPLA2; *Sections 9 & 10*, HE and MASSON staining of liver sections of *Balb*/C mouse with an abdominal injection of 100 μg MBP-*Cs*sPLA2; **b** Western blot of liver tissue of five groups of *Balb*/C mouse with P-JNK1 monoclonal antibody. Lane 1: Liver tissue of *Balb*/C mouse with an abdominal injection of PBS; Lane 2: Liver tissue of *Balb*/C mouse with an abdominal injection of 100 μg ESPs; Lane 3: Liver tissue of *Balb*/C mouse with an abdominal injection of 100 μg MBP; Lane 4: Liver tissue of *Balb*/C mouse with an abdominal injection of 50 μg MBP-*Cs*sPLA2; Lane 5: Liver tissue of *Balb*/C mouse with an abdominal injection of 100 μg MBP-*Cs*sPLA2. **c** Quantitative western blot analysis of P-JNK1. Unpaired *t*-test was applied for statistical analysis. Compared with control group, JNK signalling of experimental group, the liver tissue of *Balb*/C mouse with an abdominal injection of 50 μg MBP-*Cs*sPLA2 was activated (*t*_(4)_ = 3.298, *P* = 0.03)

Liver sections of five groups of *Balb*/C mice with hematoxylin/eosin (HE) and Masson staining were observed under a microscope. Collagen was stained blue in liver sections with Masson staining. The group of mice given an abdominal injection of 50 μg MBP-*Cs*sPLA2 showed obvious collagen accumulation compared with other groups (Fig. 4a). Western blot of the liver tissue of the five groups of mice was also done to detect whether JNK signalling pathway was activated. Consistent with the result of liver sections with Masson staining, the group of mice given an abdominal injection of 50 μg MBP-*Cs*sPLA2 showed activation of JNK signalling pathway and a clear band of P-JNK1 can be observed (Fig. 4b). Relative quantitative western blot analysis of P-JNK1 was performed to compare the difference between experimental group and control group (AlphaView Software was used to adjust the gray level of western blot image). Figure 4c provides an illustration of three independent experiments. Unpaired t-tests revealed significant differences between the group injected with 50 μg MBP-*Cs*sPLA2 and the group injected with 100 μg MBP (*t*_(4)_ = 3.298, *P* = 0.03).

### Construction of MBP-*Cs*sPLA2 mutant

**Fig. 5:**
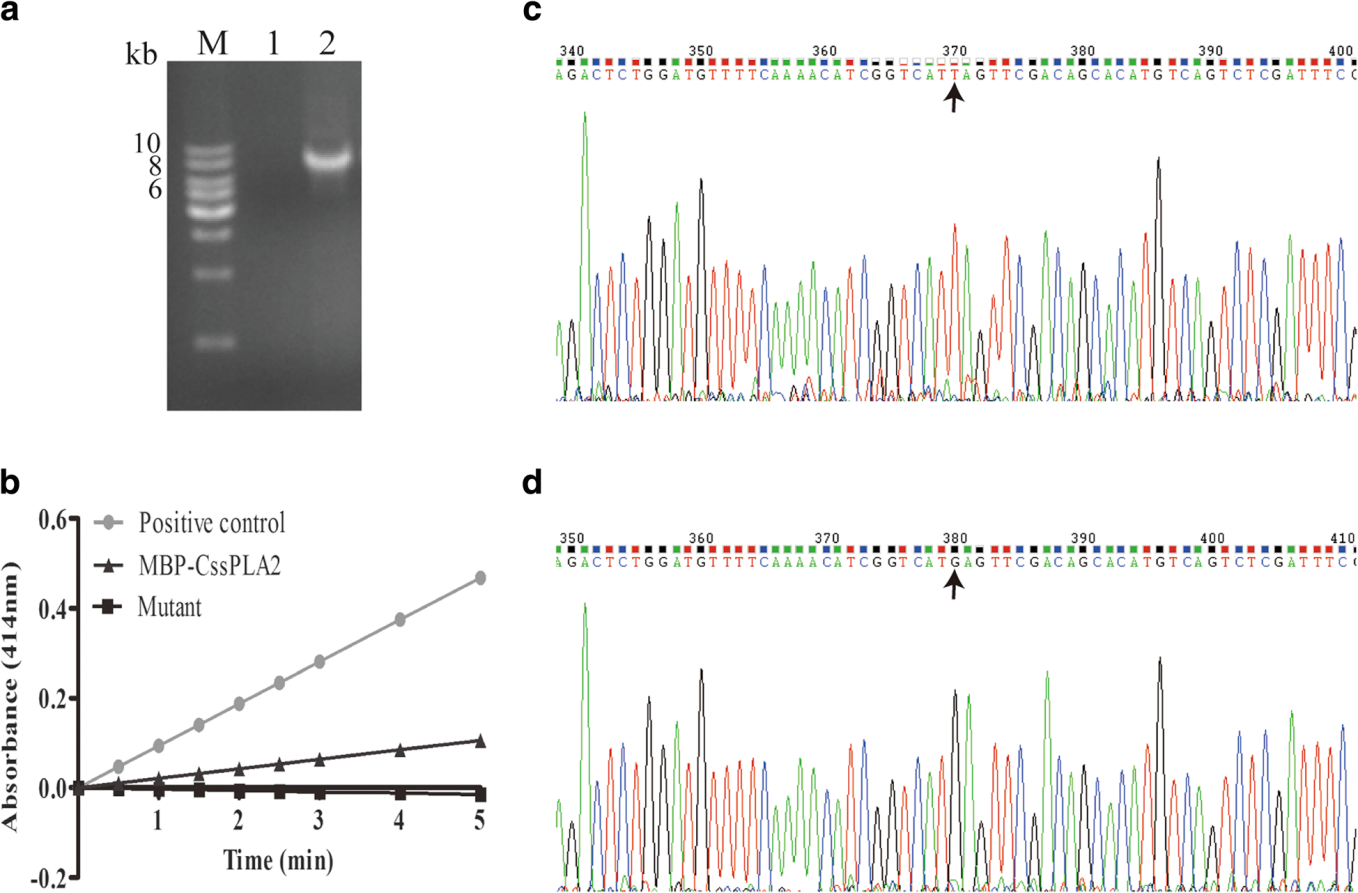
Construction of mutant of MBP-*Cs*sPLA2 without enzymatic activity. **a** PCR product of the plasmid of the mutant. Lane M: DNA molecular marker; Lane 1: ddH2O negative control; Lane 2: PCR product of mutant of MBP-*Cs*sPLA2. **b** Detection of enzymatic activity of the mutant. **c** Sequence of the mutant plasmid of *Cs*sPLA2/pMAL-c2X. **d** Sequence of the plasmid of *Cs*sPLA2/pMAL-c2X

PCR amplification with the plasmid *Cs*sPLA2/pMAL-c2X as a template was done to obtain the plasmid of the mutant (Fig. 5a). The mutant protein lost the enzymatic activity (Fig. 5b). The nucleotide sequences of the plasmid of the mutant were confirmed by DNA sequencing (Fig. 5c, d).

### JNK signalling pathway is involved in activation of LX-2 cells by MBP-*Cs*sPLA2

**Fig. 6:**
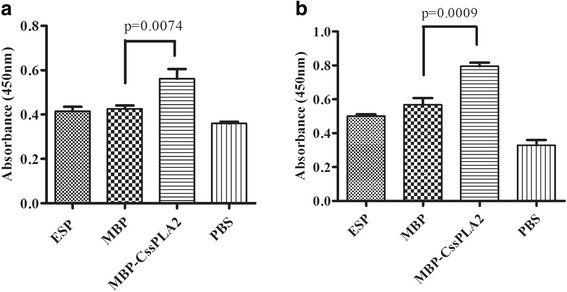
Collagen I produced by HSCs are increased after incubated with MBP-*Cs*sPLA2. The effect of MBP-*Cs*sPLA2 on activation of hepatic stellate cells and production of collagen I was evaluated by ELISA. After 36 h or 48 h, the supernatant of cells was detected by ELISA. Collagen I rabbit antibody (1:2000 dilutions) was used as the first antibody and HRP-conjugated goat anti-rabbit IgG was used as a secondary antibody (1:10,000 dilution). **a** Supernatant of cell culture after incubation for 36 h; **b** supernatant of cell culture after incubation for 48 h. Unpaired *t*-test was applied for statistical analysis

The effect of MBP-*Cs*sPLA2 on activation of hepatic stellate cells and production of collagen I was evaluated by ELISA. Either after 36 h (Fig. 6a) or 48 h (Fig. 6b), the group of cells incubated with 25 μg/ml MBP-*Cs*sPLA2 showed the largest amount of collagen I in the supernatant of cell culture. Figure 6 provides an illustration of three independent experiments. Unpaired t-tests revealed significant differences between the group incubated with 25 μg/ml MBP-*Cs*sPLA2 and the group incubated with 25 μg/ml MBP (*t*_(4)_ = 5.02, *P* = 0.0074) (Fig. 6a) and (*t*_(4)_ = 8.78, *P* = 0.0009) (Fig. 6b).

**Fig. 7:**
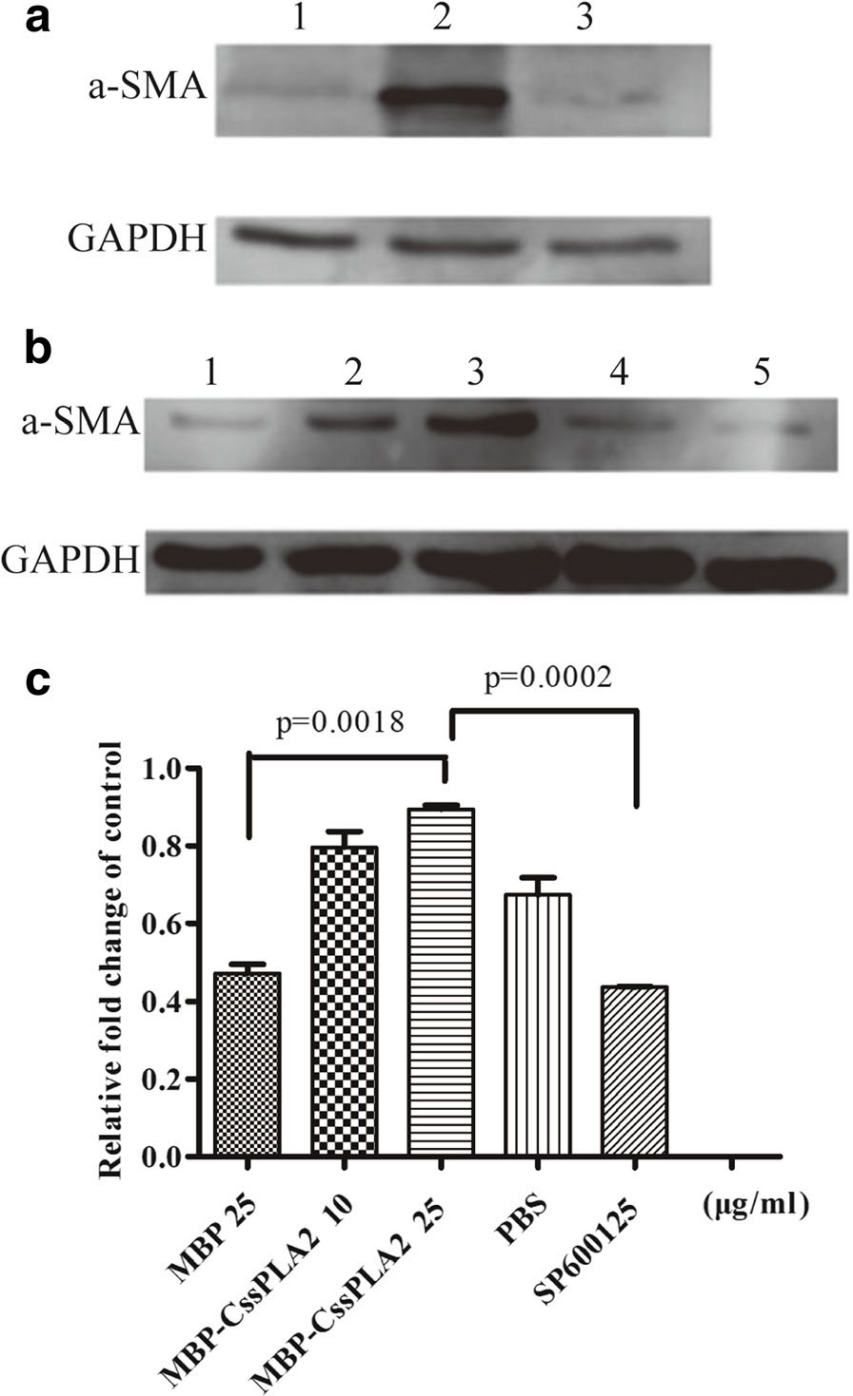
HSCs are activated after incubated with MBP-*Cs*sPLA2. The marker of the activation, α-SMA of the HSCs, was measured after incubated with 25 μg/ml MBP, 25 μg/ml MBP-*Cs*sPLA2 and PBS, respectively. Two more groups, the group of cells incubated with 10 μg/ml MBP-*Cs*sPLA2 and the group of cells incubated with both 25 μg/ml MBP-*Cs*sPLA2 and 5 μM JNK inhibitor (SP600125) were added to detect whether the activation of hepatic stellate cells by MBP-*Cs*sPLA2 was dose-dependent and whether the activation was related to activation of the JNK signalling pathway. **a** Lane 1: HSCs incubated with 25 μg/ml MBP; Lane 2: HSCs incubated with 25 μg/ml MBP-*Cs*sPLA2; Lane 3: HSCs incubated with PBS. **b** Lane 1: HSCs incubated with 25 μg/ml MBP; Lane 2: HSCs incubated with 10 μg/ml MBP-*Cs*sPLA2; Lane 3: HSCs incubated with 25 μg/ml MBP-*Cs*sPLA2; Lane 4: HSCs incubated with PBS; Lane 5: HSCs incubated with both 25 μg/ml MBP-*Cs*sPLA2 and 5 μM JNK inhibitor (SP600125). **c** Relative quantitative western blot analysis of α-SMA was performed to compare the difference between experimental group and control group. Statistical analysis were conducted with unpaired *t*-test

Western blot was also done to detect the activation of hepatic stellate cells. The marker of the activation, α-SMA of the hepatic stellate cells, was measured after incubated with 25 μg/ml MBP, 25 μg/ml MBP-*Cs*sPLA2 and PBS respectively. The hepatic stellate cells incubated with 25 μg/ml MBP-*Cs*sPLA2 showed a clear band of α-SMA (Fig. 7a).

To detect whether the activation of hepatic stellate cells by MBP-*Cs*sPLA2 was dose-dependent and whether the activation was related to activation of JNK signalling pathway, two more groups, the group of cells incubated with 10 μg/ml MBP-*Cs*sPLA2 and the group of cells incubated with both 25 μg/ml MBP-*Cs*sPLA2 and 5 μM JNK inhibitor (SP600125) were added. Western blot was done again and MBP-Cs*s*PLA2 showed a dose-dependent effect on activation of hepatic stellate cells as well as relation with activation of the JNK signalling pathway (Fig. 7b). Relative quantitative western blot analysis of α-SMA was performed to compare the difference between experimental group and control group. Figure 7c provides an illustration of three independent experiments. Unpaired t-tests revealed significant differences between the group incubated with 25 μg/ml MBP-*Cs*sPLA2 and the group incubated with 25 μg/ml MBP (*t*_(2)_ = 23.63, *P* = 0.0018), between the group incubated with 25 μg/ml MBP-*Cs*sPLA2 and the group incubated with 25 μg/ml MBP-*Cs*sPLA2 + 5 μM JNK inhibitor (*t*_(2)_ = 64.77, *P* = 0.0002).

**Fig. 8:**
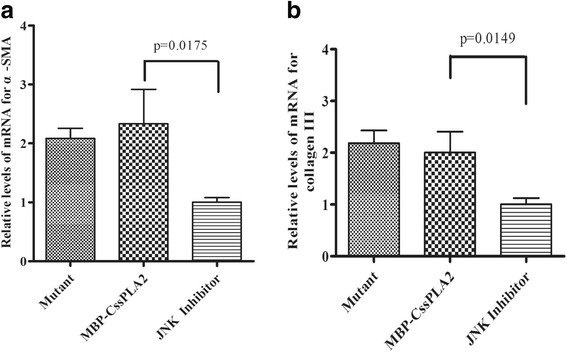
HSCs are activated after incubated with either MBP-*Cs*sPLA2 or the mutant. Quantitative RT-PCR analysis of activation markers of LX-2 was performed. The mRNA of α-SMA and collagen III were detected, respectively. The transcription levels of α -SMA and collagen III were analyzed by means of the 2^-△△Ct^ ratio, with human *β*-actin serving as the internal standard. The transcription level of α-SMA and collagen III in LX-2 cells incubated with 25 μg/ml mutant and 25 μg/ml MBP-*Css*PLA2 was similar, with no statistical significance, while that of LX-2 cells incubated with both 25 μg/ml MBP-*Cs*sPLA2 and 5 μM JNK inhibitor (SP600125) was lower than that of cells incubated with 25 μg/ml MBP-*Css*PLA2. Unpaired *t*-test was applied to compare the difference between experimental group and control group. **a** The relative levels of mRNA for α-SMA of three groups of LX-2 cells incubated with 25 μg/ml mutant, 25 μg/ml MBP-*Css*PLA2 and both 25 μg/ml MBP-*Cs*sPLA2 and 5 μM JNK inhibitor (SP600125), respectively (*t*_(4)_ = 3.905, *P* = 0.0175). **b** Relative levels of mRNA for collagen III of three groups of LX-2 cells incubated with 25 μg/ml mutant, 25 μg/ml MBP-*Css*PLA2 and both 25 μg/ml MBP-*Cs*sPLA2 and 5 μM JNK inhibitor (SP600125), respectively (*t*_(4)_ = 4.095, *P* = 0.0149)

To determine whether the activation of hepatic stellate cells by MBP-*Cs*sPLA2 was related to the enzymatic activity of the protein or not, the hepatic stellate cells were divided into three groups and incubated with 25 μg/ml mutant, 25 μg/ml MBP-*Css*PLA2, and 25 μg/ml MBP-*Cs*sPLA2 + 5 μM JNK inhibitor (SP600125), respectively. Quantitative RT-PCR analysis of activation markers of LX-2 was performed. The mRNA of α –SMA and collagen III were detected, and the difference between the group incubated with the mutant and the group incubated with MBP-*Cs*sPLA2 was of no statistical significance, while the difference between the group incubated with MBP-*Cs*sPLA2 and the group incubated with both 25 μg/ml MBP-*Cs*sPLA2 and 5 μM JNK inhibitor (SP600125) was significant: *t*_(4)_ =3.905, *P* = 0.0175 (Fig. 8a) and *t*_(4)_ = 4.095, *P* = 0.0149 (Fig. 8b). Unpaired *t*-test was applied and the difference between the group incubated with the mutant and the group incubated with MBP-*Cs*sPLA2 was of no statistical significance.

**Fig. 9:**
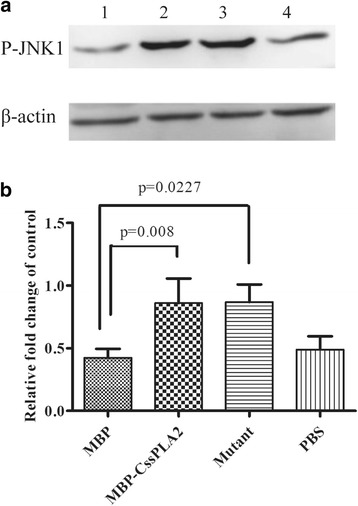
JNK signalling is activated in HSCs after incubated with either MBP-*Cs*sPLA2 or the mutant. The marker of the activation, P-JNK1 of the hepatic stellate cells, was measured after incubated with 25 μg ml MBP, 25 μg/ml MBP-*Cs*sPLA2, 25 μg/ml mutant and PBS respectively. **a** Lane 1: HSCs incubated with 25 μg/ml MBP; Lane 2: HSCs incubated with 25 μg/ml MBP-*Cs*sPLA2; Lane 3: HSCs incubated with 25 μg/ml mutant; Lane 4: HSCs incubated with PBS. **b** Quantitative western blot analysis of P-JNK1. Compared with MBP, both MBP-*Cs*sPLA2 and the mutant could activate JNK signalling. There was no significant difference between MBP-*Cs*sPLA2 and the mutant in phosphorylation of JNK1. Unpaired *t*-test was applied for statistical analysis

Western blot was done to detect the activation of JNK signalling in HSCs incubated with MBP-*Cs*sPLA2 and the mutant. The marker of activation, phospho-JNK1 of HSCs, was measured after incubated with 25 μg/ml MBP, 25 μg/ml MBP-*Cs*sPLA2, 25 μg/ml mutant and PBS, respectively. Both HSCs incubated with 25 μg/ml MBP-*Cs*sPLA2 and that incubated with 25 μg/ml mutant showed a clear band for P-JNK1 (Fig. 9a). Relative quantitative western blot analysis of P-JNK1 was performed to compare the difference between experimental group and control group. Figure 9b provides an illustration of three independent experiments. Unpaired t-tests revealed significant differences between the group incubated with 25 μg/ml MBP-*Cs*sPLA2 and the group incubated with 25 μg/ml MBP (*t*_(4)_ = 4.899, *P* = 0.008), between the group incubated with 25 μg/ml mutant and the group incubated with 25 μg/ml MBP (*t*_(4)_ = 3.605, *P* = 0.0227).

## Discussion

ESPs are continuously released by adult *C. sinensis* and contribute to interactions between the parasite and the host [19]. sPLA2 is a protein secreted by *C. sinensis* and is a component of *Cs*ESPs. After incubated with *Cs*ESPs for 24 h, LX-2 cells could be activated. It’s proved that *Cs*ESPs play an important role in liver fibrosis caused by clonorchiasis. Cell proliferation was detected by methyl thiazolyltetrazolium (MTT) assay when LX-2 cells were incubated with 50 μg/ml of CsESPs [20]. The phospholipase A2 has been well known for its function in liver fibrosis and inhibition of tumor cells. It’s suggested by the present findings that ASB14780, an IVA-PLA2 inhibitor, could be effective for the treatment of hepatic fibrosis [21]. PLA2 is an abundant component of snake venom. Bothropstoxin-I, a Lys49-phospholipase A2, which from *Bothrops jararacussu* venom was proved to cause cell death for both human and murine tumor cell lines by inducing apoptosis or necrosis [22]. In the present study, we have expressed the recombinant protein MBP-*Cs*sPLA2, which is water-soluble with good enzymatic activity. *Balb*/C mice were given an abdominal injection of MBP-*Cs*sPLA2 to detect whether the protein could activate hepatic stellate cells in vivo, and cause accumulation of collagen which leads to liver fibrosis. Hepatic stellate cells were also incubated with the protein MBP-*Cs*sPLA2 to detect the activation of the cells in vitro*.* It was proved that MBP-*Cs*sPLA2 could activate hepatic stellate cells and cause accumulation of collagen both in vivo and in vitro. This result was in accordance with the finding that the deficiency of mouse PLA2 could attenuate hepatic fibrosis formation [14]. The mutant without enzymatic activity was constructed to investigate the mechanism of activation of HSCs by MBP-*Cs*sPLA2, while it was found that activation of HSCs was not related to the enzymatic activity of the protein. To clarify whether JNK signalling pathway is involved and activated during the process, JNK inhibitor (SP600125) was applied. SP600125 is a broad spectrum JNK inhibitor for JNK1, JNK2 and JNK3 with IC50 of 40 nM, 40 nM and 90 nM, respectively; 10-fold greater selectivity against MKK4, 25-fold greater selectivity against MKK3, MKK6, PKB, and PKCα, and 100-fold selectivity against ERK2, p38, Chk1, GFR etc. In cell free assay, IC50 value is 40/40/90 nM (JNK1/2/3). However, in a cell based assay, the recommended concentration of SP600125 is 5–10 μM. In Jurkat T cells, SP600125 inhibits the phosphorylation of c-Jun with IC50 of 5 μM to 10 μM [23]. It was found that JNK signalling pathway activation had activated the hepatic stellate cells. Western blot showed that both MBP-*Cs*sPLA2 and the mutant could activate JNK signalling.

The spatial structure of *Cs*sPLA2 is complex. Therefore, it was difficult to express the protein in water-soluble form with enzymatic activity. The recombinant protein *Cs*sPLA2 was expressed by pET-28a vector as insoluble inclusion bodies and renatured by dilution and dialysis [12]. We also tried other prokaryotic vectors, such as pET-30a, pET-26b, pET-32a and pGEX-4T-1, but all the products were inclusion bodies, without enzymatic activity. Finally, the vector pMAL-c2X was chosen and water-soluble protein was expressed with good enzymatic activity. pMAL-c2X is a prokaryotic vector with a MBP (maltose binding protein) tag, which can promote the solubility of proteins. Either GST or TRX can be used for soluble expression of protein, while the MBP is much more effective [24, 25].

The sn-2 ester bond of phospholipids is hydrolysed by phospholipase A2 enzyme through a nucleophile water molecule and catalytic histidine. The histidine conformation is critical for PLA2 to hydrolyse the phospholipids [13]. The protein itself is an enzyme, and therefore, there are two ways for it to work. It works by its enzymatic activity or by activating the cell signalling pathway. The construction of a mutant was to identify which way the protein works. The mutant was the same as the protein MBP-*Cs*sPLA2 except for one amino acid difference. The 192nd amino acid of the mutant is asparagine instead of histidine, which is the most important amino acid related to the catalytic ability of the enzyme. The pathogenesis of liver fibrosis involves extracellular matrix protein deposition, including collagen I and collagen III, and requires activation of HSCs. JNK signalling pathway is related to HSCs activation. When JNK activity was blocked by the inhibitor SP600125, HSCs activity was inhibited, with reduced proliferation and less expression of α-smooth muscle actin (α-SMA) [15].

Liver fibrosis, a worldwide medical problem characterised by excessive deposition of extracellular matrix (ECM) proteins, is a scarring process caused by chronic liver injury [26, 27]. The predominant hepatic cells in the liver tissue contributing to excessive ECM deposition are HSCs [28]. As a stable and an unlimited source of human HSCs, LX-2 cell line is widely used for studying human liver fibrosis [29]. When quiescent HSCs are stimulated by liver insults, the lipid droplets are lost and a new myofibroblastic phenotype is obtained, with increased expression of ECM proteins and α-SMA [30]. The LX-2 cells were activated by the recombinant protein MBP-*Cs*sPLA2 both in vitro and in vivo.

## Conclusions

*Cs*sPLA2, as one component of *Cs*ESPs*,* can activate HSCs resulting in accumulation of collagen in vivo, which could be the reason of liver fibrosis and it can increase the level of a-SMA in hepatic stellate cells in a dose-dependent manner in vitro, which is the characteristic of activation of HSCs. The activation of hepatic stellate cells by *Cs*sPLA2 is related to activation of the JNK signalling pathway instead of the enzymatic activity of the protein, which could provide a promising strategy to interrupt the process of liver fibrosis caused by infection of *Clonorchis sinensis.*

## Abbreviations

α-SMA: α-smooth muscle actin
CCA: Cholangiocarcinoma.
*Css*PLA2: Secreted phospholipase A2 of *Clonorchis sinensis*
ECM: Extracellular matrix
ESPs: Excretory and secretory products
HSCs: Hepatic stellate cells
MBP: Maltose binding protein
*s*PLA2: Secreted phospholipase A2
